# Genome Editing for Plasmodesmal Biology

**DOI:** 10.3389/fpls.2021.679140

**Published:** 2021-06-02

**Authors:** Arya Bagus Boedi Iswanto, Rahul Mahadev Shelake, Minh Huy Vu, Jae-Yean Kim, Sang Hee Kim

**Affiliations:** ^1^Division of Applied Life Sciences (BK21 Four Program), Plant Molecular Biology and Biotechnology Research Center, Gyeongsang National University, Jinju, South Korea; ^2^Division of Applied Life Sciences, Gyeongsang National University, Jinju, South Korea

**Keywords:** plasmodesmata, CRISPR/Cas, genome editing, plant stress, crop engineering

## Abstract

Plasmodesmata (PD) are cytoplasmic canals that facilitate intercellular communication and molecular exchange between adjacent plant cells. PD-associated proteins are considered as one of the foremost factors in regulating PD function that is critical for plant development and stress responses. Although its potential to be used for crop engineering is enormous, our understanding of PD biology was relatively limited to model plants, demanding further studies in crop systems. Recently developed genome editing techniques such as Clustered Regularly Interspaced Short Palindromic Repeats/CRISPR associate protein (CRISPR/Cas) might confer powerful approaches to dissect the molecular function of PD components and to engineer elite crops. Here, we assess several aspects of PD functioning to underline and highlight the potential applications of CRISPR/Cas that provide new insight into PD biology and crop improvement.

## Introduction

The discovery of plasmodesmata (PD) in 1885 by Edward Tangl has revolutionized the field of plant science. PD functions as one of the vital controllers in plant growth and development (Wu et al., 2018; Vu et al., 2020). Briefly, PD are symplasmic (cytoplasm-to-cytoplasm) nanochannels between adjacent cells, approximately 50-60 nm in size. Structurally, a plasmodesma (the singular form of PD) consists of a cytoplasmic sleeve and a desmotubule (Bell and Oparka, 2011). The space between plasmalemma connecting the cytosol of adjacent cells is the cytoplasmic sleeve. Tubes of appressed endoplasmic reticulum (ER) connecting two adjacent cells are termed desmotubules. As symplasmic tunnels, PD provide pathways for transport of a range of molecules from cell-to-cell, including sugars, ions, proteins, and other essential nutrients, as well as different types of RNA molecules (Wu and Gallagher, 2012; Sager and Lee, 2018; Li et al., 2020b). Cell-to-cell movement of molecules through PD is thought to be dependent on a PD-size exclusion limit (PD-SEL), which involves several aspects, such as PD permeability, PD morphology, PD-associated proteins, and their functions (Sager and Lee, 2018). SEL is determined by the size of the largest molecules that can diffuse through PD. PD-SEL regulates the effectiveness of intracellular communication, which is required for plants to fine-tune their biological and developmental processes under various environmental circumstances (Wu et al., 2018). PD permeability is highly dynamic. The up-and-down modes of PD permeability are controlled by callose, a polysaccharide formed by callose (or β-1,3-glucan) synthase (CalS) enzymes and degraded by glucanase (BG) proteins (Zavaliev et al., 2011; Wu et al., 2018). Callose degradation increases the PD-SEL, whereas callose deposits reduce the PD-SEL. Moreover, PD morphology is considered an essential factor in intercellular transport and can range from simple, twinned or funnel to more complex forms (Oparka et al., 1999; Roberts et al., 2001; Faulkner et al., 2008; Nicolas et al., 2017; Ross-Elliott et al., 2017; Sager and Lee, 2018; Dorokhov et al., 2019).

As plasma membrane (PM)-lined channels, PD-PMs are occupied by unique membrane domains named lipid rafts, sterols- and sphingolipid-enriched microdomains. Lipid rafts provide attractive places for PD-receptor-like proteins (PD-RLPs) and PD-receptor-like kinases (PD-RLKs) to perceive signaling molecules in response to prevailing environmental stimuli (Iswanto and Kim, 2017; Iswanto et al., 2020; Vu et al., 2020). As a gatekeeper of cell trafficking, dynamic PD structure permits the cell-to-cell movement of endogenous molecules and acts as a channel for spreading disease-causing factors. Genome sequencing and proteome analyses are expanding the database of putative or partially characterized PD-related proteins from different plant species (Fernandez-Calvino et al., 2011; Kraner et al., 2017; Leijon et al., 2018). Also, some genes encoding PD-related proteins are redundant in sequence and function. In this regard, to characterize the functions of redundant genes, recent techniques such as genome editing serve as an ideal tool for generating knockout mutants, inducing randomized mutagenesis of the targeted region, or modulating transcriptional regulation.

The most popular genome-editing tool is CRISPR (clustered regularly interspaced short palindromic repeats)/Cas (CRISPR associated) for engineering plants at the DNA and RNA levels (Shelake et al., 2019a; Pramanik et al., 2021). CRISPR/Cas technology has been widely optimized for various applications in several plant species. Such applications include knockout generation, DNA insertion, DNA deletion, gene replacement, chromosome rearrangement, nucleic acid imaging, precise nucleotide substitution, epigenetic modification, pathogen detection, transcription regulation, and more. In this article, we highlight the role of PD-SEL (including PD-associated proteins) in response to multiple external stimuli. We also summarize the characterization of viral/fungal/bacterial proteins targeted at PD, along with potential genome editing tools, strategies, and techniques to understand the basics and improve agronomic traits through PD-SEL engineering.

## PD Proteins Involved in Abiotic Stress Responses

The characteristics of callose deposition in response to abiotic stresses (such as osmotic, drought, cold, heat, metal stress) have been reviewed in recent literature (Sager and Lee, 2014). Several reports have highlighted the factors that regulate callose accumulation, thereby conferring enhanced abiotic stress resistance. However, the mechanisms that connect callose-mediated cell-to-cell signaling to the perception of abiotic cues are elusive. This section highlights the PD-associated proteins that positively or negatively control PD-callose under abiotic stresses.

In Arabidopsis, callose deposition in response to salt (NaCl) stress was first characterized by Wrzaczek’s group. It has been reported that the receptor-like kinase (RLK), Cys-rich receptor-like kinase 2 (CRK2), can positively regulate the salt stress-dependent pathway in Arabidopsis (Hunter et al., 2019). CRK2-overexpressing lines showed an enhanced germination rate and root length under high salinity conditions. They also found that CRK2 relocalizes to PD after 15 min of mannitol treatment or 30 min of 150 mM NaCl treatment. Furthermore, CRK2 regulates callose deposition under salt-stress conditions by interacting with CalS1. They also highlighted that the CalS1 played an important role in PD permeability during salt stress. *cals1* mutant plants showed impairment in callose accumulation and germination deficiency under high NaCl treatment, which indicated that the phenotype of *cals1* was similar to the *crk2* mutant. The exact mechanism of salt-stress tolerance mediated by CRK2 relocalization (from PM to PD) and callose deposition is not clear because the CRK2-overexpression plants showed enhanced PD callose deposition and reduced PD permeability even without salt stress. Therefore, it can be hypothesized that CRK2 is implicated not only in callose-dependent salt-stress tolerance but may also be involved in plant growth and development irrespective of salt-stress conditions. In the same year, another study demonstrated that Qian Shou kinase 1 (QSK1) and inflorescence meristem kinase 2 (IMK2), a different class of RLKs, relocalize from the PM to PD in response to salt stress (Grison et al., 2019). The mechanism of callose-mediated salt stress tolerance depends on QSK1 phosphorylation but not on sterol or sphingolipid membrane composition. Interestingly, QKS1 and IMK2 rapidly modulate its localization from PM to PD within 1–4 min post-treatment of 400 mM mannitol and 100 mM NaCl. QSK1 is involved in callose deposition, the PD transportation pathway, lateral root density control, and root development. QSK1 overexpression displays an increased lateral root number and a slightly delayed lateral root formation compared to wild-type and mutant. It was also suggested that the relationship between callose accumulation and tolerance phenotypes observed with QSK1 overexpression was unclear.

Some metals were reported to trigger PD-associated proteins. Calreticulin is a highly conserved Ca^2+^-sequestering protein that typically resides within the ER lumen, especially in maize and *Medicago truncatula* (Baluska et al., 1999; Sujkowska-Rybkowska and Znojek, 2018). Under Aluminum stress, calreticulin protein in *M. truncatula* mycorrhizal roots was induced and colocalized with Ca^2+^ at the interface of fungal structures and in the periphery of the infected cortex cells (Sujkowska-Rybkowska and Znojek, 2018). Microscopic observations suggested that this colocalization might be required for the calcium mobilization that controls fungal accommodation inside the cortical cells and arbuscular development under Al stress conditions. However, the interaction of calreticulin and Ca^2+^ at PD needs further characterization. Interestingly, calmodulin (CaML) proteins have been found to reside at PD during flg22 treatment in Arabidopsis, which raises the possibility of CaML and calreticulin involvement in stress response (Xu et al., 2017; Wu et al., 2018). Similarly, treatment with subtoxic levels of copper and iron can severely inhibit primary root growth and interfere with the cell-to-cell movement of green fluorescence protein (GFP) (O’Lexy et al., 2018). Iron and copper alter PD permeability in roots via the regulation of callose synthases (CalS5, CalS12) and β-1,3-glucanases (BG_ppap, β-1,3-glucanase-putative; BG6, β-1,3-glucanase 6), respectively.

Wound stress results in alteration of callose accumulation via CalS1 and CalS8 (Cui and Lee, 2016). Aniline blue staining and Drop-ANd-See assay revealed no accumulation of PD callose in mutant leaves lacking CalS1/8 compared with wild-type after wounding. Genetically, CalS8 regulates PD permeability independently with PD-located protein 5 (PDLP5) upon wounding-induced reactive oxygen species (ROS) stress, while CalS1 requires PDLP5 in salicylic-dependent plasmodesmal response. Notably, CalS1 and CalS8 are suggested to localize along with the PM and PD. It remains to deciphered how CalS1 and CalS8 overexpression control PD permeability to enhance plant defense during biotic stresses. Abiotic stresses like heat and light trigger multi-layer signaling pathways that produce systemic acquired acclimation in plants. For example, a recent study reported the involvement of PD proteins (PDLP1 and 5) in propagating systemic ROS-signal waves in response to high light stress in Arabidopsis by altering the PD pore size (Fichman et al., 2021). Further studies into the role of PD-associated proteins in regulating the relay of different systemic signals triggered by various stresses may uncover novel mechanisms of plant protection. The list of PD-associated proteins involved in abiotic stresses is summarized in Table 1. Overall, examining the dynamic relocalization of PD-associated proteins from PM-to-PD and their role in long-distance signaling during abiotic stresses will help to understand new dimensions of PD biology.

**TABLE 1:** PD-associated proteins and their involvements in response to abiotic stress.

No	Plant species	PD-associated protein	Gene ID	Abiotic stimuli	References
(1)	*A. thaliana*	CRK2 (cys-rich receptor-like kinase 2)	AT1G70520	Salinity	Hunter et al., 2019
(2)	*A. thaliana*	QSK1 (Qian Shou kinase 1)	AT3G02880	Salinity and osmotic	Grison et al., 2019
		IMK2 (inflorescence meristem kinase 2)	AT3G51740		
(3)	*M. truncatula*	Calreticulin	MTR_7g080370 calreticulin	Aluminum	Sujkowska-Rybkowska and Znojek, 2018
(4)	*A. thaliana*	CalS5 (callose synthase 5)	AT2G13680	Heavy metals (iron, copper, zinc, and cadmium)	O’Lexy et al., 2018
		CalS12 (callose synthase 12)	AT4G03550		
		BG_PPAP (β-1,3-glucanase_putative)	AT5G42100		
		BG6 (β-1,3-endoglucanase)	AT4G16260		
(5)	*A. thaliana*	CalS1 (callose synthase 1)	AT1G05570	Wounding	Cui and Lee, 2016
		CalS8 (callose synthase 8)	AT3G14570		
(6)	*A. thaliana*	PDLP1 (plasmodesmata-located protein 1)	AT5G43980	High light	Fichman et al., 2021
		PDLP5 (plasmodesmata-located protein 5)	AT1G70690		

## PD Proteins Involved in Biotic Stress Responses

Several living organisms, specifically fungi, bacteria, yeast, nematodes, insects, arachnids, and weeds, interact with plants. These plant interactions with other species could be beneficial (mutualism), useful to another partner only (commensalism), or harmful to a partner (parasitism). When viruses, fungi, or bacteria attack the plants, it often causes disease due to their virulence activities. In many cases, invasion by pathogens causes plant growth retardation and significant losses in crop quality and productivity. To protect from pathogens, host plants have evolved diverse barricades and remarkable immune machinery for pathogen recognition and the activation of defense signaling modes (Jones and Dangl, 2006; Nguyen et al., 2021). However, some viral, fungal, and bacterial pathogens target PD to mediate intercellular spread in host plant cells.

## Virus-PD Protein Interactions

Viruses are neither “living” nor “non-living” and depend on host organisms to replicate and propagate, such as animals, bacteria, fungi, and plants. When viruses invade host plants, they form three major types of proteins, replication proteins (RPs), structural proteins (SPs), and movement proteins (MPs) which are classified based on their functions. RP is crucial for nucleic acid production; SP forms the outer protein shell and other units in the virions, whereas MP is employed to facilitate virus spread between host plant cells (Lefeuvre et al., 2019). The first study on plant viruses began in the 1890s; an infectious virus causing leaf spots in tobacco was characterized, *Tobacco mosaic virus* (TMV). TMV was the first virus of any host ever to be identified. So far, hundreds of plant viruses have been identified, almost all of which are infectious viruses of crop plants (Roossinck, 2010). Ten important plants viruses were ranked based on the scientific and economic importance, including TMV, *Tomato spotted wilt virus* (TSWV), *Tomato yellow leaf curl virus* (TYLCF), *Cucumber mosaic virus* (CMV), *Potato virus y* (PVY), *Cauliflower mosaic virus* (CaMV), *African cassava mosaic virus* (ACMV), *Plum pox virus* (PPV), *Brome mosaic virus* (BMV), and *Potato virus x* (PVX) (Scholthof et al., 2011). Plant viruses are transmitted from one plant to another by different modes such as seeds or pollen, vectors, grafting, or mechanical wounds (Hipper et al., 2013). Upon entry into the plant cell, viral components replicate and move from cell to cell through PD or are transported to long-distant organs through the vascular system. Plant viruses have evolved mechanisms of cell-to-cell movement, which involves the MP to facilitate intercellular trafficking of the plant viruses to and through the PD (Heinlein, 2015). Strikingly, some plant viruses encode multiple MPs, epitomized by triple gene block (TGB) proteins. Each TGB protein is involved in different stages of virus replication and cell-to-cell movement. In addition to MPs, some viral movement machinery requires additional virus-encoded proteins to deliver the viral genome. For instance, PVX also requires capsid protein (CP), whereas some potyviruses which do not encode MP require cylindrical inclusion protein for their cell-to-cell and long-distance dissemination (Carrington et al., 1998; Tilsner et al., 2013; Kumar et al., 2015). Many MP and other virus-encoded proteins are targeted to be localized at intercellular host regions such as the chloroplast, vesicles, ER, Golgi apparatus, nucleus, PM, and PD apertures. The list of plant virus-encoded proteins-targeted PD is summarized in Table 2.

**TABLE 2:** List of plants virus/bacterial/fungal-encoded proteins-targeted PD.

No.	Pathogen	Protein name	Subcellular localization	Host plant/characterized from	References
(1)	*Red clover mottle virus* (RCMV)	43-kDa	PD	Cowpea (*Vigna unguiculata*)	Shanks et al., 1989
(2)	*Tobacco mosaic virus* (TMV)	30-kDa MP	PD	Tobacco	Wolf et al., 1989
(3)	*Cowpea mosaic virus* (CPMV)	48-kDa	PD	Cowpea (*Vigna unguiculata*)	Wellink et al., 1993
(4)	*Maize streak virus* (MSV)	PV1	PD	Maize (*Zea mays* L.)	Dickinson et al., 1996
(5)	*Potato leafroll virus* (PLRV)	pr17-kDa	PD	Potato (*Solanum tuberosum* L.)	Schmitz et al., 1997
(6)	*Cucumber mosaic virus* (CMV)	3a MP	PD	Cucumber (*Cucumis sativus*)/*Nicotiana clevelandii*	Blackman et al., 1998
(7)	*Olive latent virus 2* (OLV-2)	36K	PD, cell walls, and cytoplasm	*N. benthamiana* and *N. tabacum*	Grieco et al., 1999
(8)	*Beet necrotic yellow vein virus* (BNYVV)	P42 MP	PD	*Chenopodium quinoa*	Erhardt et al., 2000
(9)	*Beet yellows virus* (BYV)	Hsp70h	PD	*Chenopodium quinoa/N. benthamiana*	Avisar et al., 2008
(10)	*Brome mosaic virus* (BMV)	3a MP	PD	*N. benthamiana*	Kaido et al., 2007
(11)	*Lettuce infectious yellows virus* (LIYV)	36-kDa (P26)	PD	Lettuce/*N. tabacum*	Stewart et al., 2009
(12)	*Turnip mosaic virus* (TuMV)	P3N-PIPO	PD	Turnip/*N. benthamiana*	Wei et al., 2010; Chai et al., 2020
(13)	*Turnip mosaic virus* (TuMV)	6K_2_	vesicle, PM, and PD	Turnip/*N. benthamiana*	Grangeon et al., 2013
(14)	*Potato mop-top pomovirus* (PMTV)	TGB3	ER, PD	*N. benthamiana*	Tilsner et al., 2010
(15)	*Bean dwarf mosaic virus* (BDMV)	BDMV-MP	PD	*N. benthamiana*	Zhou et al., 2011
(16)	*Rice stripe virus* (RSV)	NSvc4	PD	*Oryza sativa* L./*N. benthamiana*	Yuan et al., 2011; Xu and Zhou, 2012
(17)	*Rice grassy stunt virus* (RGSV)	pC6	cell wall, PD	*N. benthamiana*	Hiraguri et al., 2011; Sui et al., 2018
(18)	*Broad bean wilt virus 2* (BBWV-2)	VP37	PD	*Chenopodium quinoa*	Liu et al., 2011
(19)	*Rice transitory yellowing virus* (RTYV)	P3	Nucleus and PD	*Oryza sativa* L./*N. benthamiana*	Hiraguri et al., 2012
(20)	*Grapevine virus A* (GVA)*/grape virus B* (GVB)	p31/p36	PD	*Vitris vinifera* L./*N. benthamiana*	Haviv et al., 2012
(21)	*Rice black-streaked dwarf virus* (RBSDV)	P7-1	Nucleus, cytoplasm, and PD	*Oryza sativa* L., *Zea mays* L., *Hordeum vulgare* L., *Triticum aestivum* L./*N. benthamiana*	Sun et al., 2013
(22)	*Raspberry leaf blotch emaravirus* (RLBV)	P4	PM and PD	*Rubus/N. benthamiana*	McGavin et al., 2012; Yu et al., 2013
(23)	*Chinese wheat mosaic virus* (CWMV)	37K	PD and ER	*Triricum*, cereal plants worldwide*/N. benthamiana*	Andika et al., 2013
(24)	*Citrus psorosis virus* (V)	54K	PD	Citrus*/N. benthamiana*	Robles Luna et al., 2013
(25)	*Mirafiori lettuce big-vein virus* (MiLBVV)	54K	PD	Lettuce/*N. benthamiana*	Robles Luna et al., 2013
(26)	*Apple chlorotic leaf spot virus* (ACLSV)	50 kDa	cytoplasm and PD	Apple/*N. occidentalis*	Yoshikawa et al., 1999
(27)	*Cauliflower mosaic virus* (CaMV)	P6	PD	*N. benthamiana*	Rodriguez et al., 2014
(28)	*Pepper ringspot virus* (PepRSV)	P29	PD	*Capsicum* sp./*N. benthamiana*	Rodrigues et al., 2015
(29)	*Turnip vein-clearing virus* (TVCV)	P30	PD	Turnip/*N. benthamiana*	Mann et al., 2016
(30)	*Lettuce necrotic yellows virus* (LNYV)	P3	PD	Lettuce/*N. benthamiana*	Mann et al., 2016
(31)	*Alfalfa dwarf virus* (ADV)	P3	PD	Lucerne or alfalfa (*Medicago sativa* L.)/*N. benthamiana*	Mann et al., 2016
(32)	*Melon necrotic spot virus* (MNSV)	DGBp2	PD	Melon (*Cucumis melo* L.)/*N. benthamiana*	Genoves et al., 2011; Navarro and Pallas, 2017
(33)	*Melon necrotic spot virus* (MNSV)	p7B	ER, Golgi apparatus, and PD	Melon (*Cucumis melo* L.)/*N. benthamiana*	Genoves et al., 2011
(34)	*Capsicum chlorosis virus* (CaCV)	NSm	Cell periphery and PD	*Capsicum annuum* L. and *Solanum lycopersicum* L./*N. benthamiana*	Widana Gamage and Dietzgen, 2017
(35)	*Citrus tristeza virus* (CTV)	P23	Nucleolus, cajal bodies and PD	Citrus/*N. benthamiana*	Ruiz-Ruiz et al., 2018
(36)	*Cucurbit chlorotic yellows virus* (CCYV)	P4.9	Nucleus, cytoplasm, and PD	Cucumber (*Cucumis sativus* L.) and melon (*Cucumis melo* L.)/*N. benthamiana*	Wei et al., 2019
(37)	*Pepper vein yellows virus* (PeVYV)	P4	PD	*Capsicum* sp./*N. benthamiana*	Li et al., 2020a
(38)	*Barley stripe mosaic virus* (BSMV)	γb	Chloroplast, ER, actin filaments, and PD	Barley (*Hordeum vulgare* L.)/*N. benthamiana*	Jiang et al., 2020
(39)	*Grapevine fanleaf virus* (GFLV)	2B	PD	*Vitis vinifera* L./*N. benthamiana*	Amari et al., 2010
(40)	*Fusarium oxysporum* f. sp. *lycopersici*	Avr2 and Six5 (interaction)	PD	Tomato (*Solanum lycopersicum* L.)/*N. benthamiana*	Cao et al., 2018
(41)	*Melampsora larici-populina*	MLP37347	PD	Genus Populus/*A. thaliana*	Germain et al., 2018
(42)	*Phytophthora brassicae*	RxLR3	PD	*Brassica oleracea* L. and *Brassica sinensis* L./*N. benthamiana* and *A. thaliana*	Tomczynska et al., 2020
(43)	*Pseudomonas syringae* pv. *tomato (Pst)* DC3000	HopO1-1	PM and PD	Tomato (*Solanum lycopersicum* L.)/*N. benthamiana* and *A. thaliana*	Aung et al., 2020

Since many plant virus-encoded proteins localize to PD, it has been assumed that these symplasmic channels play a pivotal role in the viral spread. Plant viruses have evolved in several ways to achieve virulence and pathogenicity. However, PD-SEL is considered one of the main factors limiting the spread of virus infection (Kumar et al., 2015). The PD-SEL is highly linked to the callose accumulation at the edges of PD; therefore, the regulation of CalSs or BGs are depicted as the central signaling pathways to maintain intercellular trafficking via PD (Wu et al., 2018). It has been reported that increased callose accumulation at PD through the suppression of class I BG (GLU I, β-1,3-glucanase) inhibits intercellular movement of TMV, PVX, CMV in the tobacco plants. In contrast, increased PD flux by class III BG (GLU III) overexpression dilates the spread of potato virus Y^*NTN*^ (PVY^*NTN*^) in the potato plants (Iglesias et al., 2000; Bucher et al., 2001; Dobnik et al., 2013), see Table 2. The alteration of callose-mediated PD-SEL upon virus infection also involves the physical interaction between PD-associated proteins and virus-encoded proteins. A cytoplasmic receptor ankyrin repeat-containing protein 1 (ANK1) from *Nicotiana tabacum* recruited and interacted directly with TMV MP at PD, resulting in callose attenuation, subsequently enhancing the cell-to-cell movement of TMV MP (Ueki et al., 2010). In addition to PD-associated proteins, PDLP1 interacts with 2B MP from *Grapevine fanleaf virus* (GFLV) at PD, and a *pdlp1/2/3* triple mutant leads to reduced intercellular movement of GFLV (Amari et al., 2010). Besides PDLP1, PDLP5 may also be essential for the movement of other viral proteins. It has been reported that PDLP5 regulates PD permeability in a callose-dependent manner, and reduced callose accumulation in the *pdlp5* mutant exhibits increased cell-to-cell movement of TMV MP30 (Cui and Lee, 2016). However, it remains unknown whether PDLP1 regulates the cell-to-cell movement of GFLV through a callose-dependent manner, and it has not yet been explicitly verified whether PDLP5 physically interacts with TMV MP30.

In addition to PD-associated proteins, a plant-specific lipid microdomain and PD protein, *Solanum tuberosum* Remorin 1.3 (*St*REM1.3), physically interacts with PVX TGB1 protein (Raffaele et al., 2009). The overexpression of *StREM1.3* significantly inhibits the cell-to-cell movement of PVX TGB1, TMV MP30 as well as PVY Hc-Pro (Raffaele et al., 2009; Perraki et al., 2014). Another study on plant REM has shown that *Nicotiana benthamiana REM1* (*NbREM1*) is a negative regulator of the intercellular movement of *Rice stripe virus* (RSV) through the *S-*acylation suppression process (Fu et al., 2018). Although a PVX TGB2 protein interacts indirectly with a BG protein (Fridborg et al., 2003) and grain setting defect 1 (GSD1) (Gui et al., 2015), a REM protein identified from *Oryza sativa* interacts directly with OsACT1 at PD in controlling PD permeability (Gui et al., 2014). Most recent studies on plant REM indicate that the restriction of PVX spread occurs in a REM-induced callose accumulation-dependent manner and may involve the activation of salicylic acid (SA) signaling (Perraki et al., 2018; Huang et al., 2019). Overall, REM proteins from different plant species were reported to be implicated in callose deposition at PD, a key mechanism in plant development and stress responses. Therefore, REM interaction with viral components could be targeted by genome editing or transgenic technology for imparting viral-stress tolerance depending on the negative or positive effect on the viral spread, respectively.

In structure, PD represents membrane-lined canals that provide a suitable compartment for plant receptors to perceive diverse environment-related stimuli. Some of the plant receptors are predominantly localized or recruited at PD in response to abiotic and biotic stresses (Vu et al., 2020). In the case of viral infection, host plants have evolved an antiviral defense mechanism, namely RNA interference (RNAi) mediated by small interfering RNA (siRNA) (Borges and Martienssen, 2015). This RNAi moves from cell to cell through PD to overcome virus infectivity (Smith et al., 2007). However, viruses also develop viral suppressors of RNA silencing (VSR) to target multiple parts of the RNAi machinery (Csorba et al., 2015). In the recent study of virus-related PD-RLKs, BARELY ANY MERISTEM 1 and 2 (BAM1 and BAM2) are essential for the cell-to-cell movement of RNAi whereby they interact with C4 protein from TYLCV (Rosas-Diaz et al., 2018) and the viral silencing suppressor P19 from *Tomato bushy stunt virus* (TBSV) at PD (Garnelo Gomicronmez et al., 2021). However, the role of BAM1 and BAM2 in callose-mediated PD closure is still elusive. In addition to PD-PM protein, SYNAPTOTAGMIN A (SYTA)- an ER-PM contact site protein- can be recruited at PD to facilitate the cell-to-cell movement of *Turnip vein-clearing virus* (TVCV) MP (Levy et al., 2015). SYTA also interacts with the TMV MP and PD localization signal (PLS) of TMV MP and other virus-encoded proteins from *Cabbage leaf curl virus* (CaLCuV). The suppression of *SYTA* leads to reduced cell-to-cell movement of TMV MP, inhibited the systemic spread of CaLCuV, *Turnip mosaic virus* (TuMV), and TVCV, and disrupted PD targeting of TMV PLS (Lewis and Lazarowitz, 2010; Uchiyama et al., 2014; Yuan et al., 2016; Yuan et al., 2018). However, it remains unknown whether SYTA-mediated viral movement occurs in a callose-mediated PD closure-dependent manner or not. A recent study highlighted the importance of phosphorylatable amino acid residues of CMV MP in symptom development and PD localization (Sáray et al., 2021). Investigating such new aspects will shed light on virus-plant host interactions in detail and provide potential clues toward designing novel crop protection strategies in the future.

## Fungal/Bacterial-PD Protein Interactions

Like pathogenic viruses, plant pathogenic fungi and bacteria cause different diseases that hinder crop quality and productivity. The following plant pathogenic fungi and bacteria have been listed based on their scientific and economic importance. The list of pathogenic fungi includes *Magnaporthe oryzae*, *Botrytis cinerea*, *Puccinia* spp., *Fusarium graminearum*, *Fusarium oxysporum*, *Blumeria graminis*, *Mycosphaerella graminicola*, *Colletotrichum* spp., *Ustilago maydis*, and *Melampsora lini* (Dean et al., 2012). The list of pathogenic bacteria includes *Pseudomonas syringae* pathovars, *Ralstonia solanacearum*, *Agrobacterium tumefaciens*, *Xanthomonas oryzae* pv. *oryzae*, *Xanthomonas campestris* pathovars, *Xanthomonas axonopodis* pathovars, *Erwinia amylovora*, *Xylella fastidiosa*, *Dickeya* (*dadantii* and *solani*), *Pectobacterium carotovorum*, and *Pectobacterium atrosepticum* (Mansfield et al., 2012). Like viruses, pathogenic fungi and bacteria have also evolved sophisticated machinery to invade their host plants. The most common approach for invasion among pathogenic fungi and bacteria is to deploy various effector proteins that can target and modulate PD channels, thus activating various processes in host plants (Lee and Lu, 2011). A hemibiotrophic rice blast fungus *M. oryzae* utilizes invasive hyphae to exploit PD channels (Kankanala et al., 2007) and spread to neighboring cells through PD to expand its vicinity, possibly by delivering an effector Pathogenicity toward Weeping Lovegrass (PWL2) protein (Khang et al., 2010). In addition to *M. oryzae*, the effectiveness of fungal growth from one cell to a neighboring cell is mainly controlled by the attenuation of callose deposition at PD in which a single fungal mitogen-activated protein kinase (MAPK), PmK1, is involved (Sakulkoo et al., 2018). *Melampsora larici-populina* causes rust disease and severe problems in the genus Populus plants and other family *Salicaceae* plants. *M. larici-populina* is grouped into biographic plant-parasites that secrete an assortment of effectors to determine host cell colonization. A recent study indicates that one of the effectors from *M. larici-populina*, MLP37347, is located at PD (Germain et al., 2018). Even though MLP37347 is targeted to PD, there is no unequivocal evidence showing that MLP37347 effector regulates PD function during *M. larici-populina* infection. It will be interesting to explore the role of the MLP37347 effector in correlation with PD biology. Other effectors from *F. oxysporum*, Avr2 and Six5, have been reported to interact at PD. This interaction is required to manipulate PD apertures, allowing Avr2 to move from one cell to neighboring cells. The presence of Six5 is required for Avr2 cell-to-cell movement through PD, whereas without Avr2, the Six5 effector alone is not sufficient to alter PD permeability. This experiment indicates that to trigger PD opening upon *F. oxysporum* infection, the interaction between Avr2 and Six5 effectors in host cells is required (Cao et al., 2018).

To manipulate the immunity and physiology of host plants, pathogenic fungi and bacteria not only secrete effectors but also target them into PD aperture or other host interiors. Like viruses, it can be assumed that some fungi or bacteria effectors target PD and interact directly with PD-associated proteins to regulate symplasmic continuity. Recently, two pathogen effectors, RxLR3 from *Phytophthora brassicae* and HopO1-1 from *P. syringae*, were reported to localize at PD and interact with PD proteins (Aung et al., 2020; Tomczynska et al., 2020). RxLR3 targets CalS1, CalS2, and CalS3 to control symplasmic trafficking through callose turnover at PD (Tomczynska et al., 2020). Unlike the RxLR3 effector, HopO1-1 physically associates with other PD proteins, such as PDLP5 and PDLP7, to hamper their stability (Table 3). The destabilization of PDLP5 and PDLP7 proteins upon HopO1-1 infection leads to enhanced symplasmic conductivity (Aung et al., 2020). It has been shown that PDLP5 is involved in the immune response upon bacterial infection through maintaining callose accumulation at PD (Lee et al., 2011; Cui and Lee, 2016). Furthermore, the mechanism of HopO1-1-enhanced PD permeability seems to be PDLP5/PDLP7-regulated callose accumulation-dependent. In addition to HopO1-1, recent studies reported that several effectors from *P. syringae* not only localized at PD, but they also moved symplastically between the cells through these channels (Kang et al., 2021; Li et al., 2021). It was also suggested that the intercellular movement of effectors is PD permeability dependent manner (Li et al., 2021). However, the molecular linkage between the intercellular movement of effectors and PD regulation is still poorly understood.

**TABLE 3:** PD-associated proteins and their interactions with virus/fungal/bacterial proteins.

No	Plant species	PD-associated protein	Gene ID	(Virus/fungal/bacterial) protein	References
(1)	*A. thaliana*	CalS3/GSL12	AT5G13000	(*Phytophthora brassicae*) RxLR3	Tomczynska et al., 2020
(2)	*A. thaliana*	PDLP5	AT1G70690	(*Pst* DC3000) HopO1-1	Aung et al., 2020
(3)	*A. thaliana*	PDLP7	AT5G37660	(*Pst* DC3000) HopO1-1	Aung et al., 2020
(4)	*A. thaliana*	BAM1	AT5G65700	(TYLCV) C4	Rosas-Diaz et al., 2018
(5)	*A. thaliana*	BAM1	AT5G65700	(TBSV), P19	Garnelo Gomicronmez et al., 2021
(6)	*A. thaliana*	BAM2	AT3G49670	(TYLCV) C4	Rosas-Diaz et al., 2018
(7)	*A. thaliana*	BAM2	AT3G49670	(TBSV) P19	Garnelo Gomicronmez et al., 2021
(8)	*Solanum tuberosum*	StREM1.3	NP_001274989/102577743	(PVX) TGBp1	Raffaele et al., 2009; Perraki et al., 2014
(9)	*A. thaliana*	PDLP1	AT5G43980	(GFLV) 2B	Amari et al., 2010
(10)	*A. thaliana*	Calreticulin	AT1G09210	(TMV) MP30	Chen et al., 2005
(11)	*A. thaliana*	SYTA	AT2G20990	(TMV) 30K, (CaLCuV) MP, (TVCV) MP, and (SqLCV) MP	Lewis and Lazarowitz, 2010; Uchiyama et al., 2014; Levy et al., 2015; Yuan et al., 2016, 2018

## Genome Editing Tools

Recent advancements in genome engineering tools based on CRISPR/Cas systems have opened new doors to fine-tune the plant genome at all layers of the central dogma (Pramanik et al., 2021). Another major advantage of CRISPR-based tools is the ability to customize a strategy to precisely edit the redundant genes or simultaneously edit multiple homologs (Wang et al., 2019; Hong et al., 2020). CRISPR-based tools have been employed in the editing of PD-related genes in recent times (Rosas-Diaz et al., 2018), but their real potential is yet to be explored for manipulating PD biology. In the following sections, we present and discuss the CRISPR/Cas tools and their future applications to investigate fundamental aspects of PD biology or PD engineering for stress management strategies.

**FIGURE 1:**
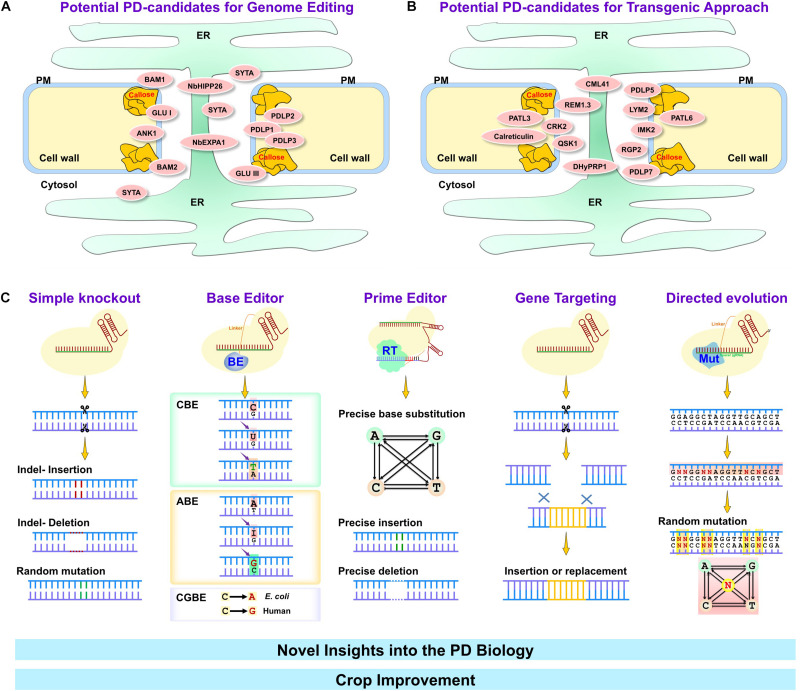
Major genome editing techniques for crop improvement through plasmodesmal engineering. Many PD-associated proteins are involved in a variety of environmental stresses (abiotic and biotic stresses). Depending on the impact on stress mechanism, particular PD-associated proteins can be classified as negative **(A)** or positive **(B)** regulators. PD-associated proteins that negatively regulate stress tolerance in plants (summarized in panel **A**) can be targeted using genome editing tools such as simple knockout by CRISPR/Cas, base editor, prime editor, gene targeting and directed evolution tools **(C)**. On the other hand, PD-associated proteins that positively regulate stress tolerance in plants (summarized in panel **B**) may provide the easiest way to overexpress them by transgenic approach. Targeting PD-associated proteins by genome editing or transgenically possess potential avenues to improve crop quality and productivity. ER, Endoplasmic reticulum; PM, plasma membrane; BAM1 and 2, barely any meristem 1 and 2; GLU I and III, β-1,3-glucanase class I and III; ANK1, ankyrin repeat-containing protein; SYTA, Synaptotagmin A; NbHIPP26, *Nicotiana benthamiana* heavy metal-associated isoprenylated plant protein; NbEXPA1, *N. benthamiana* α-expansin 1; PDLP1, 2, 3, 5, and 7, Plasmodesmata-located protein 1, 2, 3, 5, and 7; PATL3 and 6, patellin 3 and 6; CML41, calmodulin-like protein 41; REM1.3, Remorin 1.3; CRK2, Cys-rich receptor-like kinase 2; QSK1, Qian Shou kinase 1; DHyPRP1, double hybrid proline-rich protein 1; LYM2, lysin motif domain-containing glycosylphosphatidylinositol-anchored protein 2; IMK2, inflorescence meristem kinase 2; RGP2, reversibly glycosylated polypeptide 2.

The first report demonstrating the potential of CRISPR/Cas components for genome editing was published in 2012 (Jinek et al., 2012). Since then, tremendous progress has been made in developing novel CRISPR-based tools (Figure 1). The primary CRISPR/Cas tool consists of two components comprising a nuclease enzyme (Cas) and a programmable RNA guide (gRNA) complementary to the target DNA. Cas enzyme bound with scaffold-fused gRNA (sgRNA) recognizes the target site followed by a short recognition motif called protospacer adjacent motif (PAM). The protein-RNA-DNA complex formation leads to the generation of DNA double-strand breaks (DSBs) at desired sites in the complex genome, and endogenous DNA repair pathways make precise or error-prone DNA modifications. The Cas9 and Cas12a (Cpf1) are the most commonly applied Cas enzymes for mutant creation in different organisms (Shelake et al., 2019b). Various Cas variants and orthologs have been characterized to maximize the editing scope and different PAM specificities. Simultaneous targeting of multiple loci in the genome is a significant advantage of CRISPR-based tools compared to other genome engineering methods.

The use of CRISPR/Cas in homologous recombination-based gene targeting (HR-GT) has demonstrated the potential to improve gene-targeting efficiency through precise DSB induction if a donor template is provided to promote the homology-directed repair (HDR) pathway (Capdeville et al., 2021). The HDR-based GT mostly occurs in dividing cells and desired HR-GT products are often mixed with additional indels (insertions/deletions) due to preferred non-homologous end joining (NHEJ). To address this issue, partial catalyzed (nickase, D10A, or H840A) or fully deactivated (dead D10A together with H840A) Cas9 nuclease is engineered for delivering the effector molecules to the target locus for many applications beyond simple DSB-mediated knockout generation (Adli, 2018). Primary tools based on the fusion of effector molecules with nCas9 or dCas9 include base editors that introduce base substitutions without the need for HDR, DSBs, or donor templates (Komor et al., 2016; Gaudelli et al., 2017). A recent addition to the CRISPR toolbox is the prime editor, which needs a template (Anzalone et al., 2019). Although the prime editor tool can introduce customized changes (small insertions or deletions, all 12 base substitutions) at the targeted genomic locus, optimization for plant use is desirable in the near future. Several *in silico* and *in vivo* protocols are being devised for target site selection, validation of gRNAs, appropriate choice of CRISPR tool for the desired application, and suitable delivery strategies depending on the species (Huang et al., 2021). Overall, several features like simple design, high precision, efficiency, lower cost, choice of versatile tools, and a broad range of targeting in the genome have enabled the wider adoption of CRISPR/Cas technology for various purposes in plants.

### Genome Editing of PD-Related Genes

Despite the discovery of PD in the nineteenth century, precise knowledge about PD structure and function is still elusive. Although PD operating mode remains challenging to understand, applications of contemporary techniques are revealing their novel facets. Broadly, CRISPR-based technologies can be applied in PD research with bidirectional aims. The first direction is the understanding of PD biology, and secondly, targeting PD-related proteins for the development of stress-tolerant crops. The choice of the CRISPR tool predominantly depends on the possible outcome. As discussed earlier, CRISPR tools may produce a variety of genetic modifications- for example, simple knockout, base substitution, precise insertion/deletion/replacement, strong/weak allele generation, epigenetic modulation, transcriptional regulation, and chromosomal rearrangements (Shelake et al., 2019a). We describe the potential of CRISPR tools for exploiting plasmodesmal biology in two parts: understanding the basics of PD functioning and their modulation for stress management.

### Genome Editing for Understanding PD Biology and Crop Improvement

The PD interactome can be roughly divided into three parts depending on their direct or indirect role in PD formation and functioning. Group 1 consists of the actual players that form the PD structure itself; the second group involves the molecules that regulate the PD SEL. The third group contains the molecules trafficking through PD. The interplay between the molecules from these three groups is crucial not only to plant physiology and development but also to plant stress responses and environmental signals (Azim and Burch-Smith, 2020). On the one hand, long-distance trafficking of soluble molecules and defense signals occurs through PD. On the other hand, pathogens also hijack the PD cell-to-cell movement machinery to spread from infected to non-infected plant parts. Therefore, the PD-PM interface is at the forefront of the battle between pathogens and plant defense molecules.

Considerably, several studies have uncovered different facets of PD-mediated spread of viruses and plant defense signaling molecules such as siRNAs. For example, the C4 protein of TYLCV primarily interacts with proteins implicated in plant defense, ubiquitination, and translation from host tomato plants (Kim et al., 2016). Recent reports showed that the RLK homologs (BAM1 and BAM2) act as a positive regulator of siRNA spread through PD (Rosas-Diaz et al., 2018). CRISPR-mediated double knockout mutants (*bam1 bam2*) were generated, confirming the redundant role of BAM1 and BAM2 in promoting the cell-to-cell spread of RNAi. Also, this study suggested the C4 interaction halts the BAM1/2 function and eventually the spread of RNA silencing. In the follow-up study, another viral protein, P19 from TBSV, was demonstrated to interact with BAM1/2 in a similar fashion like C4, indicating that BAM1 and BAM2 are good candidates for CRISPR targeting of C4/P19-interacting domains to develop geminiviral-resistant plants (Garnelo Gomicronmez et al., 2021). Overall, CRISPR-mediated genome editing of PD-related genes is valuable to explore their function and provides attractive potential candidates from the PD interactome to edit and develop biotic and abiotic stress tolerance (Table 4).

**TABLE 4:** Genetic engineering strategies for modulating PD-associated proteins.

No	Gene name	Gene ID	Reported study		Proposed genetic engineering technique		Purpose	References
			
			KO/KD	OE	CRISPR/Cas9	OE		
(1)	*BAM1*	AT5G65700	Inhibits RNAi movement	Promotes RNAi movement	o (modifying C4/P19-interacting domain)	o	Biotic stress tolerance (TYLCV, TBSV)	Rosas-Diaz et al., 2018; Garnelo Gomicronmez et al., 2021
(2)	*BAM2*	AT3G49670	Inhibits RNAi movement	nd	o (modifying C4/P19-interacting domain)	o	Biotic stress tolerance (TYLCV, TBSV)	Rosas-Diaz et al., 2018; Garnelo Gomicronmez et al., 2021
(3)	*CRK2*	AT1G70520	S	R	x	o	Abiotic stress tolerance (salinity)	Hunter et al., 2019
(4)	*IMK2*	AT3G51740	nd	R	x	o	Abiotic stress tolerance (salinity and drought)	Grison et al., 2019
(5)	*QSK1*	AT3G02880	S	R	x	o	Abiotic stress tolerance (salinity and drought)	Grison et al., 2019
(6)	*PDLP1,2,3*	AT5G43980, AT1G04520 and AT2G33330	R	nd	o	x	Biotic stress tolerance (GFLV)	Amari et al., 2010
((7)	*PDLP5*	AT1G70690	S	R	x	o	Biotic stress tolerance (*Pst* DC3000, *Psm* ES4326, TMV and CMV)	Lee et al., 2011; Lim et al., 2016; Aung et al., 2020
(8)	*PDLP7*	AT5G37660	S	nd	x	o	Biotic stress tolerance (*Pst* DC3000 and *Psm* ES4326)	Aung et al., 2020
(9)	*LYM2*	AT2G17120	S	nd	x	o	Biotic stress tolerance (*Botrytis cinerea*)	Faulkner et al., 2013
(10)	*GLU I*	–	R	S	o	x	Biotic stress tolerance (TMV, PVX and CMV)	Iglesias et al., 2000; Bucher et al., 2001
(11)	*StREM1.3*	NP_001274989/102577743	S	R	x	o	Biotic stress tolerance (PVX)	Raffaele et al., 2009; Perraki et al., 2014
(12)	*RGP2*	AT5G15650	nd	R	x	o	Biotic stress tolerance (TMV)	Zavaliev et al., 2010
(13)	*ANK1* and *ANK2*	AAK18619/AAN63819	R	S	o	x	Biotic stress tolerance (TMV)	Ueki et al., 2010
(14)	*GLU III*	KC437380	nd	S	o	x	Biotic stress tolerance (*potato virus Y^*NTN*^*)	Dobnik et al., 2013
(15)	*DHyPRP1*	AT4G22470	S	R	x	o	Biotic stress tolerance (*Pst* DC3000 and *Botrytis cinerea*)	Li et al., 2014
(16)	*CML41*	AT3G50770	S	R	x	o	Biotic stress tolerance (*Pst* DC3000)	Xu et al., 2017
(17)	*NbEXPA1*	NbS00007680g0013.1	nd	S	o	x	Biotic stress tolerance (TuMV)	Park et al., 2017
(18)	*NbHIPP26*	Niben101Scf02621g04026.1	R	nd	o	x	Biotic stress tolerance (PMTV)	Cowan et al., 2018
(19)	*PATL3* and *PATL6*	AT1G72160 and AT3G51670	S	R	x	o	Biotic stress tolerance (*alfalfa mosaic virus*, AMV)	Peiro et al., 2014
(20)	*Calreticulin*	AT1G09210	nd	R	x	o	Biotic stress tolerance (TMV)	Chen et al., 2005
(21)	*SYTA*	AT2G20990	R	nd	o	x	Biotic stress tolerance (TMV, CaLCuV, TVCV and SqLCV)	Lewis and Lazarowitz, 2010; Uchiyama et al., 2014; Yuan et al., 2018

*KO, knock out; KD, knock down; OE, overexpression; S, susceptible; R, resistant; nd, not determined; O, modification expected for the positive effect.*

The symbiotic interaction between host plant-PD and nitrogen-fixing microbes is another research area that needs to be explored. Recent work has shed some light on the molecular dialog between the host plant and associated microbes confirming that PD regulation is a key early event for establishing the symbiotic legume plant-microbe association (Gaudioso-Pedraza et al., 2018). The PD-localized β-1,3-glucanase from *Medicago truncatula* MtBG2 promoted the symplasmic connectivity, thereby facilitating the nodule formation. The increased PD permeability (Complainville et al., 2003) or higher number of PD pores (Schubert et al., 2013) substantially increased nodule number in *M. truncatula* and *Casuarina glauca*, respectively. Also, some tetraspanin proteins like TET3 from Arabidopsis (Fernandez-Calvino et al., 2011), PvTET3, and PvTET6 from the common bean were reported to be localized at the PD-PM interface during nodule formation with rhizobia (Jimenez-Jimenez et al., 2019), suggesting their direct role in symplasmic interaction through PD regulation and cellular trafficking. Thus, the use of CRISPR tools in altering PD may help to promote the positive interaction of symbiotic association of nitrogen-fixing microbes and host plants.

The new set of plant breeding techniques, collectively known as new plant breeding technologies (NPBT), includes the concept of grafting wild-type onto genetically modified (GM) rootstock (Langner et al., 2018). The proper combination of scion and rootstock is advantageous to develop improved crop traits. The bi-directional interaction between rootstock and scion involves exchanging all three major macromolecules (DNA, RNA, and protein) through the PD. A recent report showed that even the genomes could transfer horizontally via organelle travel during the remodeling of PD and vascular connection at the root-scion junction (Hertle et al., 2021). Previous reports have successfully used transgenic rootstocks to transfer transgene-mediated traits to the wild-type scion parts- for example, the development of CMV resistance in tomato (Bai et al., 2016), Pierce’s disease resistance in grape (Dandekar et al., 2019), PPV resistance in plum (Sidorova et al., 2021), and increased nitrogen levels in walnut overexpressing an ammonium transporter gene (Liu et al., 2021). In such studies, engineering of PD trafficking and the use of transgene-free CRISPR techniques to attain desired traits would be highly desirable because the non-transgenic genome editing approach may easily avoid the GM issues and related-regulatory hurdles.

## Conclusion and Perspective

PD-mediated symplasmic transport permits cell-to-cell communication in multicellular plants, regulating the harmonized physiological growth and development during environmental stresses. Even though the dynamic nature of PD allows surprisingly high intercellular transport of molecules, PD plasticity makes it challenging to establish the regulatory mechanisms of PD functioning. In this regard, advanced techniques like genome editing and high-resolution microscopy are promising to solve the mysteries around PD structure and function. The primary goal of crop improvement is to design climate-resilient varieties with superior traits. The crucial role of the PD interactome in plant defense is now well-known. The use of genome editing in PD engineering has a vast potential to improve molecule transport for higher nutrition quality for human health, to protect plants against biotic and abiotic stresses, to design improved symbiotic interactions for plant nutrition, and to enhance grafting-based strategies for crop improvement.

## Author Contributions

ABBI and RMS designed the manuscript structure. ABBI, RMS, and MHV wrote the manuscript. J-YK and SHK designed the manuscript structure and edited the manuscript. All authors contributed to the article and approved the submitted version.

## Conflict of Interest

The authors declare that the research was conducted in the absence of any commercial or financial relationships that could be construed as a potential conflict of interest.

## Abbreviations

ACLSV: *Apple chlorotic leaf spot virus*
ACMV: *African cassava mosaic virus*
ADV: *Alfalfa dwarf virus*
ANK1: *Ankyrin 1*
BAM: BARELY ANY MERISTEM
BBWV-2: *broad bean wilt virus 2*
BDMV: *Bean dwarf mosaic virus*
BG: β -1,3-glucanase
BMV: *Brome mosaic virus*
BNYVV: *Beet necrotic yellow vein virus*
BSMV: *Barley stripe mosaic virus*
BYV: *Beet yellows virus*
CaCV: *Capsicum chlorosis virus*
CaLCuV: *Cabbage leaf curl virus*
CalS: callose synthase
CaML: calmodulin
CaMV: *Cauliflower mosaic virus*
CCYV: *Cucurbit chlorotic yellows virus*
CMV: *Cucumber mosaic virus*
CP: capsid protein
CPMV: *Cowpea mosaic virus*
CPsV: *Citrus psorosis virus*
CRISPR: clustered regularly interspaced short palindromic repeats
CRK2: cys-rich receptor-like kinase 2
CTV: *Citrus tristeza virus*
CWMV: *Chinese wheat mosaic virus*
DHyPRP1: double hybrid proline-rich protein
DSB: double-strand break
ER: endoplasmic reticulum
EXPA1: expansin 1
GFLV: *Grapevine fanleaf virus*
GM: genetically modified
GSD1: grain setting defect 1
GVA: *Grapevine virus A*
GVB: *Grapevine virus B*
HDR: homology-directed repair
HIPP26: heavy metal-associated isoprenylated plant protein 26
HR-GT: homologous recombination-based gene targeting
IMK2: inflorescence meristem kinase 2
LIYV: *Lettuce infectious yellows virus*
LNYV: *Lettuce necrotic yellows virus*
LYM2: Lysin motif domain-containing glycosylphosphatidylinositol-anchored protein 2
MAPK: mitogen-activated protein kinase
MiLBVV: *Mirafiori lettuce big-vein virus*
MNSV: *Melon necrotic spot virus*
MP: movement protein
MSV: *Maize streak virus*
NHEJ: non-homologous end joining
NPBT: new plant breeding technologies
OLV-2: *Olive latent virus 2*
PAM: protospacer adjacent motif
PATL: Patellin
PD: plasmodesmata
PDLP: plasmodesmata-located protein
PeVYV: *Pepper vein yellows virus*
PLRV: *Potato leafroll virus*
PLS: PD localization signal
PM: plasma membrane
PMTV: *Potato mop-top pomovirus*
PPV: *Plum pox virus*
PepRSV: *Pepper ringspot virus*
PVX: *Potato virus x*
PVY: *Potato virus y*
PWL: Pathogenicity toward Weeping Lovegrass
QSK1: Qian Shou Kinase 1
RBSDV: *Rice black-streaked dwarf virus*
RCMV: *Red clover mottle virus*
REM: Remorin
RGP2: reversibly glycosylated polypeptide 2
RGSV: *Rice grassy stunt virus*
RLBV: *Raspberry leaf blotch emaravirus*
RLK: receptor-like kinase
RLP: receptor-like protein
RNAi: RNA interference
ROS: reactive oxygen species
RP: replication protein
RSV: *Rice stripe virus*
RTYV: *Rice transitory yellowing virus*
SA: salicylic acid
SEL: size exclusion limit
sgRNA: scaffold-fused gRNA
siRNA: small interfering RNA
SP: structural protein
SYTA: SYNAPTOTAGMIN A
TBSV: *Tomato bushy stunt virus*
TGB: triple gene block
TMV: *Tobacco mosaic virus*
TSWV: *Tomato spotted wilt virus*
TuMV: *Turnip mosaic virus*
TVCV: *Turnip vein-clearing virus*
TYLCF: *Tomato yellow leaf curl virus*
TYLCV: *Tomato yellow leaf curl virus*
VSR: viral suppressors of RNA.
